# Non-functioning Well Differentiated Endocrine Carcinoma of the Pancreas

**DOI:** 10.4021/gr2009.01.1266

**Published:** 2009-11-20

**Authors:** Tadashi Terada

**Affiliations:** Department of Pathology, Shizuoka City Shimizu Hospital, Miyakami 1231 Shimizu-Ku, Shizuoka 424-8636, Japan. Email: piyo0111jp@yahoo.co.jp

**Keywords:** Pancreas, Endocrine carcinoma, Histopathology, Immunohistochemistry

## Abstract

The author reports a typical but rare case of non-functioning well differentiated endocrine carcinoma of the pancreas. A 67-year-old man was admitted to our hospital because of abdominal pain. No hormone-related symptoms were recognized. He has no familiar history of pancreatic neoplasms. Various imaging modalities including US, CT and MRI revealed a tumor of the pancreatic body. Distal pancreatectomy and splenectomy were performed. A solid well demarcated tumor was present in the pancreatic body. Peripancreatic lymph nodes showed marked swelling suggestive of metastases. Immunohistyochemically, tumor cells were positive for cytokeratin, synaptophysin, neuron-specific enolase, and CD56; they were negative for chromogranin, gastrin, glucagon, somatostatin, pancreatic polypeptide, and vasoactive intestinal polypeptide. The pathological diagnosis was non-functioning well differentiated endocrine carcinoma of the pancreas.

## Introduction

Pancreatic neoplasms are classified into exocrine and endocrine neoplasms [1, 2]. The majority of pancreatic neoplasm is exocrine neoplasm such as ductal adenocarcinoma. Endocrine neoplasm accounts for 1-2% of all pancreatic neoplasms [1-3]. Pancreatic endocrine neoplasms were classified into endocrine microadenoma, well-differentiated pancreatic endocrine neoplasm, poorly differentiated endocrine carcinoma, and mixed endocrine carcinoma [1, 2]. The well differentiated pancreatic endocrine neoplasms were further categorized into functional and nonfunctional ones. The author herein reports a typical but rare case of non-functioning well differentiated pancreatic endocrine carcinoma.

## Case report

**Figure 1:**
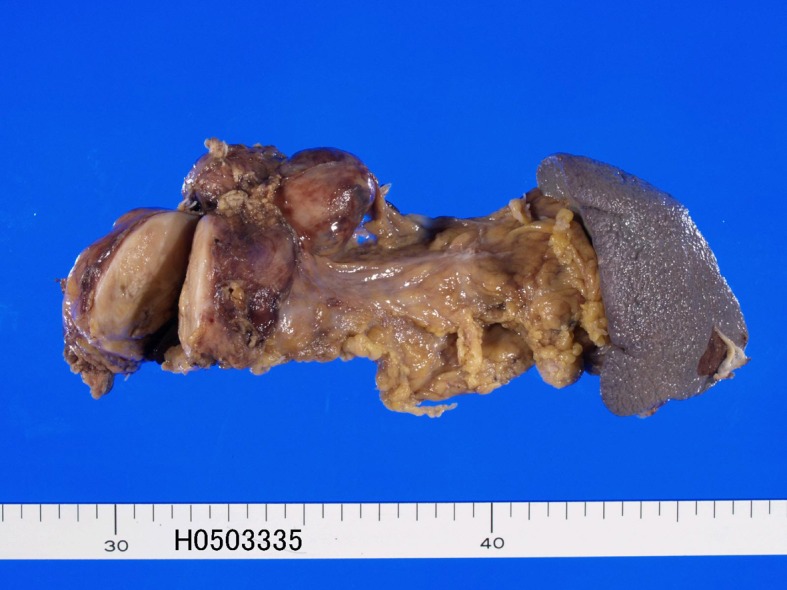
Gross features of resected pancreas. A well defined tumor measuring 60 x 55 x 50 mm is recognized (left). Lymph node metastasis is also seen (left upper).

**Figure 2:**
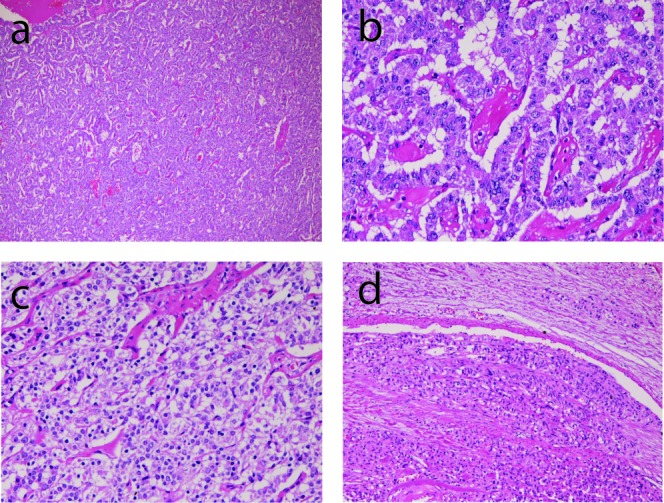
Microscopic findings of the tumor. (a) very low power view of the tumor. Tumor cells are arranged in a trabecular pattern. HE, x20. (b) tumor cells are arranged in a trabecular pattern. The nuclei show ‘salt and pepper’ appearances. HE, x200. (c) clear cell change of tumor cells. HE, x200. (d) vascular invasion of tumor cells. HE, x100.

A 67-year-old man was admitted to our hospital because of abdominal pain. No hormone-related symptoms were recognized. He denied a familiar history of MEN type I and Hippel-Lindau syndrome. Various imaging modalities including US, CT and MRI revealed a tumor of the pancreatic body. Distal pancreatectomy and splenectomy were performed. Grossly, a solid well-defined tumor measuring 60 x 55 x 50 mm was present in the pancreatic body (Fig. 1). Peripancreatic lymph nodes showed marked swelling suggestive of metastases (Fig. 1). Histologically, tumor cells with hyperchromatic nuclei were arranged in a trabecular patten (Fig. 2a, 2b). Clear cell change was recognized in some areas (Fig. 2c). The nuclei of tumor cells showed ‘salt and pepper’ appearances (Fig. 2b, 2c), and mitotic figures were present in 5 per 10 high power fields. Invasive features, and vascular and perineural invasions were recognized in the periphery of the tumor (Fig. 2d). No ductal element was recognized. The peripancreatic lymph nodes showed metastases. The spleen was devoid of carcinoma cells.

An immunohistochemical study was performed using Dako Envision methods (Dako Corp. Glostrup, Denmark), as previously described [4, 5]. The antibododies used were anti-cytokeratin (AE1/3, Dako), anti-cytokeratin (polyclonal wide, Dako), carcinoembrionic antigen (CEA) (polyclonal, Dako), chromorgranin (DAK-A3, Dako), synaptophysin (polyclonal, Dako), neuron-specific enolase (NSE) (BBS/NC/VI-H14, Dako), CD56 (MOC-1, Dako), insulin (polyclonal, Dako), glucagon (polyclonal, Dako), gastrin (polyclonal, Dako), somatostatin (polyclonal, Dako), pancreatic polypeptide (polyclonal, Dako), and vasoactive intestinal polypeptide (polyclonal, Dako).

**Figure 3:**
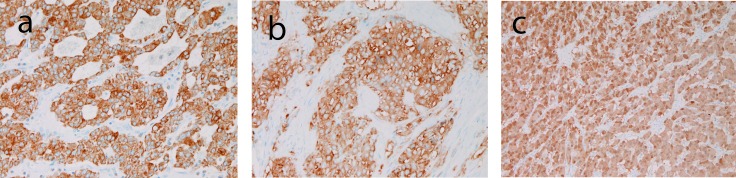
Immunohistochemical findings of the tumor. (a) tumor cells are positive for cytokeratin. Immunostaining, x200. (b) Tumor cells are positive for synaptophysin. Immunostaining, x200. (c) Tumor cells are positive for CD56. Immunostaining, x200.

Immunohistyochemically, tumor cells were positive for cytokeratin (Fig. 3a), synaptophysin (Fig. 3b), neuron-specific enolase, and CD56 (Fig. 3c); they were negative for chromogranin, gastrin, glucagon, somatostatin, pancreatic polypeptide, and vasoactive intestinal polypectide.

The pathological diagnosis was non-functioning well differentiated endocrine carcinoma of the pancreas. At the 36 months post-operative follow-up, the patient was alive with liver metastases.

## Discussion

The present case is typical pancreatic well differentiated endocrine tumor. The trabelular arrangement and ‘salt and pepper’ nuclei of tumor cells are typical for this neoplasm. Further, the present tumor was immunohistochemically positive for neuroendocrine markers (synaptophysin, NSE and CD56), highly suggesting that the present is an endocrine tumor. The absence of ductal element indicates that the present tumor is not mixed endocrine tumor [5, 6]. Clear cell change of tumor cells, as seen in the present case, has been reported to be present in pancreatic endocrine tumors and it is due to lipid deposition [7]. In addition, the present case showed infiltrative growth, perineural invasion, vascular permeation, and metastases, indicating that the present case is malignant, namely the well differentiated pancreatic endocrine carcinoma.

The present case clinically lacked hormone-related paraneoplasmic syndrome. In addition, the present endocrine carcinoma was immunohistochemically negative for insulin, glucagon, gastrin, pancreatic polypeptide, and vasoactive intestinal polypeptide. These observations indicate that the present case is non-functional pancreatic endocrine carcinoma. In addition, the absence of familiar history of MEN type I and Hippel-Lindau syndrome suggests that the present tumor is not familiar cancer syndrome but a sporadic non-functional endocrine carcinoma.

The incidence of non-functional endocrine carcinoma is less than 1% of all pancreatic neoplasms [8]. Clinically, presenting symptoms are non-specific, such as nausea and abdominal pain, in non-functional endocrine tumor [1-3].

The present case was characterized by abdominal pain. This contrasts with functional endocrine tumor such as insulinoma, gastrinoma, glucagonoma, PPoma and VIPoma, which shows specific hormone-related paraneoplasmic syndrome [1-3]. The present case lacked this syndrome. The age of the patients with pancreatic endocrine tumor ranges from 40 to 60 ages, the mean being 58 years. The present case was 67 years. Male to female ratio is 1:1 [1-3]. The treatment is operation. Adjuvant chemotherapy and radiation may be useful [1-3].

The prognosis of pancreatic endocrine tumors depends on tumor size, histology, and tumor stages, but the 5-year survival after pancreatic resection is 65% and 10-year survival is 45% [8]. The present case was alive at the 36th month follow-up after operation.

The pathogenesis of pancreatic endocrine tumors is unknown. Accumulating studies have suggested that pancreatic endocrine tumors are derived from ductal cells or ductal stem cells [8-10]. However, in MEN type I, the pancreatic endocrine tumors arise from islets of Langerhans [1, 2]. Much more studies are required to determine the original cells of pancreatic endocrine tumors.
